# Effect of boric acid on poly vinyl alcohol- tannin blend and its application as water-based wood adhesive

**DOI:** 10.1080/15685551.2020.1826124

**Published:** 2020-10-05

**Authors:** Ravindra V. Gadhave, Vineeth S.K., Pritam V. Dhawale, Pradeep T. Gadekar

**Affiliations:** Department of Polymer and Surface Engineering, Institute of Chemical Technology, Mumbai, India

**Keywords:** Tannin, poly vinyl alcohol, crosslinking, polymer blend, wood adhesive, adhesion

## Abstract

The work presented here focusses on developing adhesive by blending tannin and polyvinyl alcohol (PVA) in water. To furthermore enhance the properties crosslinking is carried by using boric acid at varying concentrations. Presence of free hydroxyl groups in PVA and tannin acts as a site for crosslinking reaction. The empty p orbital of trivalent boron atom attracts nucleophilic hydroxyl groups of PVA and tannin, hence are expected to form crosslinks. The interaction of boric acid with the blend was confirmed by FTIR spectra studies. The acidic pH favoured the reaction and its effects were observed by increase in viscosity and glass transition temperature (Tg). Films cased with the crosslinked blend demonstrated less hydrophilic behaviour from water contact angle test also increment in pencil hardness value and stress-bearing capacity. Adhesive performance properties like wet tack and time-dependent tensile lap shear strength on softwood and hardwood specimens were evaluated. The crosslinking-enhanced cohesion by reducing the free volumes between the chains and due to this, enhancement in tensile strength on bonded wood substrates was observed. Overall, it was found that the adhesive prepared by crosslinking PVA/tannin blend with boric acid is suitable for wood adhesive application.

## 1. Introduction

In the wood adhesive sector, formaldehyde-based thermosetting resins, such as urea-formaldehyde (UF), phenol-formaldehyde (PF), resorcinol-formaldehyde (RF) or melamine-formaldehyde (MF) were the conventionally used adhesives [1]. Toxicity of formaldehyde emission from these systems and the use of other organic solvents were identified for the cause of cancer and hence the demand decreased for these systems [2–5]. Moreover, while formulation toxic plasticizers are also added for getting better adhesive properties [6–9]. Thus, the state of art and research on new bio-based alternatives to conventional petrochemicals showed tremendous increase in the past few decades [10–12]. The main attractions of these bio-based alternatives such as cellulose, starch, tannin, lignin, soy protein etc., that are bio-degradability, sustainability, low cost with the possibilities for further modification [13–16]. Although formaldehyde-based systems are carcinogenic, efforts are being devoted to develop durable adhesives using phenolic substitutes [17]. Investigators have utilized renewable and natural polymers to replace phenol in PF type adhesives [18], and to reduce phenolic content with extenders/modifiers [19]. One such example of natural phenolic compound having number of active hydroxyl and carboxyl group is tannin [20].

Tannin is an abundant natural polymer and is relatively inexpensive. Condensed tannins as eco-friendly raw material for resin production are excellent renewable alternatives to overcome drawbacks of petroleum-derived wood adhesives [21,22]. The reactions of tannins which are known as auto condensation, take place in the absence of external hardener and involve opening of the rings under alkaline or acidic conditions [23]. The main woody plant species from which tannin can be obtained are mimosa, quebracho and radiata pine [21,24]. The rate of auto condensation varies depending on the source. Mimosa and quebracho tannin are slower-reacting tannin compared with pine and pecan nut tannin which has rapid auto condensation reactions [25,26]. Problems of tannin glues are largely those of rapid reactions between tannins and formaldehyde, short adhesive activity, poor water resistance, high viscosity, high dosage, component variation and easy chemical reactions. If technical challenges can be overcome, there is great commercial potential for tannin as a phenolic substitute in wood adhesives [27,28].

**Figure 1.**
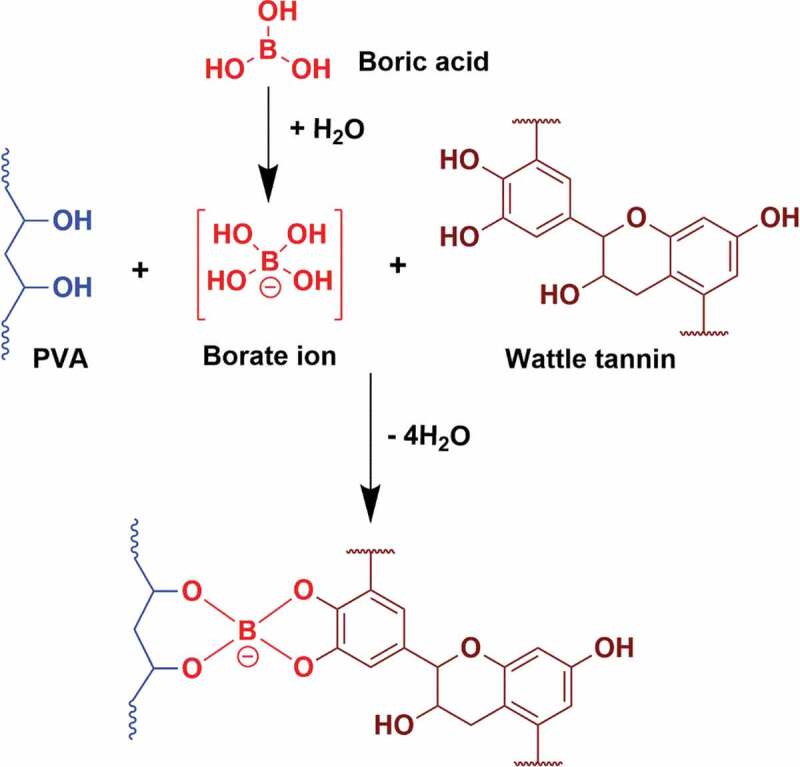
Possible reaction of tannin and PVA with boric acid

Couple of attempts have been made to improvise the poor surface bonding of natural tannin-based adhesive. Blending of tannin with other adhesive can be one such method to improve the adhesive properties of tannin. It is seen that cornstarch-wattle tannin adhesive without formaldehyde made for interior plywood application, exhibited comparable properties to the commercial PF adhesive with enhanced mechanical properties [22]. In another work where polyvinyl acetate (PVAc) was incorporated to the natural tannin adhesive to increase viscosity of tannin adhesive for surface bonding [29]. Twenty percent of PVAc contents of hybrid adhesives showed better bonding than the commercial natural tannin adhesive with a higher level of wood penetration. In the case of polyvinyl alcohol (PVA), which is one of the most common water-based adhesive, cross-linking is a common strategy to improve the performance for adhesive applications [30]. PVA has been cross-linked with cross-linking agents, such as weak Lewis acid, zirconium chloride, boric acid, aluminium chloride, titanium chloride, etc. The cross-linking helps to improve the mechanical properties and water stability of PVA based adhesives. Boric acid cross-linker for PVA adhesives have shown enhanced thermal, mechanical and adhesive performance properties [31]. In addition to enhancing mechanical and adhesion properties, it was found that boric acid can be a preservative to tannin-based wood adhesive against fungal attack [32,33]. Boric acid forms strong bonds with free hydroxyl groups due to presence of vacant d-orbital in boron, which causes it to react with various nucleophiles to form complexes. Since PVA and tannin also have free hydroxyl groups, it is expected that similar crosslinking reaction can take place if they are blended. The mechanism of crosslinking reaction of boric acid with PVA and tannin is shown in Figure 1.

From various literatures on wood adhesives, PVA and tannin have potential application in wood adhesive sector. Although PVA and tannin are used in wood adhesives both separately, the properties of adhesive formed by blending of PVA and tannin remains scant. Hence to fill the research gap, the work investigates the property of adhesive prepared by blending PVA and tannin. PVA/tannin blend adhesives are further modified by crosslinking with boric acid. Water is used as a solvent in preparing the blend, as both PVA and tannin are soluble in water. Various characterizations have been performed to evaluate physical, thermal, mechanical and adhesive properties of the prepared samples. It is expected that boric acid crosslinks with the blend and can contribute positively for enhancing performance properties of wood adhesive.

## 2. Materials and methods

### 2.1. Materials

About 86.5–89% hydrolyzed PVA powder with molecular weight of 99,000–105,000 g/mol was purchased from Kuraray Cooperative Limited, India. Boric acid with 98% pure and melting point of 171°C was purchased from Sigma-Aldrich. Wattle tannin (fine powder, brown colour, 8.5% moisture content) was supplied by Sri Guru Chemicals, Kolkata. Distilled water, which was prepared in our lab was used throughout the whole experiment.

### 2.2 Preparation method

A flat bottom reactor equipped with a stirrer and a condenser was used in preparing PVA/tannin blend. As per Table 1, PVA and tannin were blended in water. The reactor was placed in a water bath having programmable temperature control. The blending is done by heating and under stirring at 200 rpm. As the temperature reached 60°C, boric acid was added to the system and raised the temperature to 95°C. Temperature was maintained as it reached 95°C and the reaction was continued for 2.5 h. Throughout the reaction, condenser allows water to remain in the system, making no changes in the solid content of the adhesive. A blank blend without boric acid was also prepared for comparative study. The blended samples were then cooled to 28°C and then made available for further characterization. The blended samples were labelled as per Table 1.

**Table 1.** Table showing the formulation of various blended samples

	Blend sample name	PVA wt.%	Tannin wt.%	Boric acid wt.%	Water wt.%
**Formulation**	**Tan P blank**	13	5	0	82.0
	**Tan P B 0.5%**	13	5	0.5	81.5
	**Tan P B 1%**	13	5	1	81.0

### 2.3 Hydroxyl value determination of tannin

Hydroxyl value of the tannin was determined by ASTM D 1957–63 (Acetylation method). To 5 mL of pyridine-acetic anhydride solution (3:1), 2 gm of wattle tannin is taken in a 250-mL Erlenmeyer flask and mixed. Reflex condenser is inserted into the Erlenmeyer flask and then heated for 1 h. 10 mL of distilled water is then added through the condenser and again heated for 10 minutes and then kept for cooling at room temperature. To the sample, 25 mL of n-butyl alcohol and a few drops of phenolphthalein indicator solution is added. Finally, the sample is titrated against 0.5 N alcoholic KOH solution till a faint pink colour appears and the value of KOH required is noted as the end point. For calculation, a blank is also made in a similar manner without tannin. Hydroxyl value is calculated as per the following formula:

$$Hydroxylvalue=\frac{NormalityofKOH\times VolumeofKOH\left(mL\right)\text{\textbackslash{}break}\times 56.1}{weightoftannin\left(g\right)}$$

The result is reported in the unit mg of KOH/g of sample.

### 2.4 Fourier transform infra-red spectroscopy (FTIR)

FTIR spectra of the samples were determined by a PerkinElmer FTIR spectrum 100 instruments in the range 4000–650 cm^−1^ with 24 scans at a resolution of 4 cm^−1^. The samples were kept in a moisture-free atmosphere 24 h prior to the testing.

### 2.5 Viscosity and pH

A Brookfield DV1 Viscometer was used for measuring the viscosities of adhesive samples at 28°C temperature and 20 rpm. The pH of the samples was determined by a digital pH meter CL 54 + from Toshcon Industries Ltd., India. Viscosity and pH measurements were repeated for three times to minimize the error.

### 2.6 Contact angle

To study the hydrophilic/hydrophobic nature of the samples, contact angle was made using distilled water over the sample films and was measured using Rame-Hart Goniometer, Germany.

### 2.7 Differential scanning calorimetry (DSC)

The glass transition temperature (Tg) of the white glue samples were obtained from Differential scanning calorimetry (DSC) experiment using Perkin Elmer instrument Q 100 DSC. Before the analysis films of the samples were cased over a Teflon sheet. The films measured 200 µm were then properly dried at room temperature. Inert atmosphere in the DSC cell was maintained by purging nitrogen gas thought the experiment at a flow rate of 40 mL/min. Film samples were heated from 20 to 300°C at a rate of 20°C min^−1^. Transitions were determined from the thermogram obtained for the respective samples.

### 2.8 Pencil hardness test

The pencil hardness of films made from the adhesive samples were tested according to the standard, ASTM D 3363, using QHQ-A portable pencil hardness tester. Scratches were made on the casted films of adhesive samples using lead pencils of varying hardness and the values are then recorded.

### 2.9 Ultimate stress of films

A universal testing machine (UTM) of Tinius Olsen 5ST was used for measuring the ultimate stress of the adhesive films.

### 2.10 Wet tack

Wet tack data relate to the binding properties of an adhesive. To study wet tack of prepared samples, Probe tack analyzer from Rohit Instruments, Pidilite industries, R & D lab, Mumbai, India was used. The instrument consists of a metallic cylinder and a polished surface. 2 mg of the samples to be analyzed is poured in such a way it completely spread over the surface. The metallic cylinder is then allowed to approach the surface with sample. The whole system is automated and the speed at which cylinder moves is kept as 5 mm/min. Once after the cylinder gets in contact with the surface containing sample, the cylinder retraces back. The resistance offered by the adhesive against its upward motion is termed as wet tack and have the unit as gram force (gf). The test was conducted at temperature of 28–30°C, with relative humidity of 65–70% and this data is saved. Wet tack data is repeated 5 times for reducing the error.

### 2.11 Tensile strength

To study the effect of bonding strength of the samples with the plywood (ply), laminate (lam) and Canarium wood specimens, tensile lap shear strength was measured using a universal testing machine (UTM) Tinius Olsen H25KT, Mumbai, India. Test method was according to ASTM D 906. The plywood (ply), laminate (lam) and Canarium wood specimens were cleaned to remove dust particles prior to test. A constant amount of the prepared samples was spread over onto ply and lam specimen of 50 mm × 50 mm area and 25 mm × 25 mm for canarium wood specimens, making sure that the sample properly spread over the entire area. The adhesive applied wooden samples were oriented parallel and are then held by grips with total load of 5 kg cylinder + 2.5 kg arm. The bonded specimens were then loaded to the UTM apparatus and test was run at a pulling rate of 5 mm/min. The test was conducted after a fixed time interval of 2 h, 4 h, 6 h and 24 h so that the bond development time can be investigated. To minimize error, five bonded test specimens were tested and the standard deviation were reported. The whole test was conducted at temperature of 28–30°C, with relative humidity of 65–70%.

## 3 Results and discussion

### 3.1 Hydroxyl value determination of tannin

To find out the possibility of crosslinking reaction with tannin, it should have free hydroxyl groups. The hydroxyl value determination as per ASTM D 1957–63 (Acetylation method), helps to quantify the free hydroxyls in tannin. From the titration, it was seen that 14.87 mL of KOH was required to change the solution to faint pink colour, which is the end point. Hence, hydroxyl value determination of tannin is found to have a value of 208.6 mg of KOH/g, which shows a good value, making the crosslinking reaction possible with tannin also. Since tannin is blended with PVA and then crosslinked, the hydroxyl value of the blend was not calculated.

### 3.2 Fourier transform infra-red spectroscopy (FTIR)

**Figure 2.**
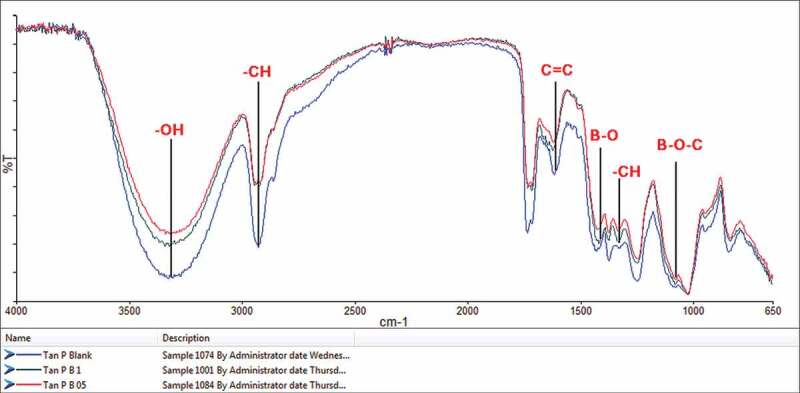
Overlaid FTIR spectra of all the samples

The FTIR spectra of the blank blend and PVA/tannin blends with 0.5 and 1 wt.% boric acid are displayed in Figure 2. The broad band around 3300 cm^−1^ corresponds to the hydroxyl (–OH) stretching and hydrogen-bonded hydroxyls [6,7,9], due to the presence of free hydroxyl groups in PVA/tannin blend. It is seen that the intensities of the peak reduce for both the blends with boric acid compared to the blank sample and is reflected in almost all the peaks. This can be due structural changes occurred during the crosslinking reaction as per Figure 1, where the free – OH groups are used up by boric acid and making changes in the hydrogen-bonded hydroxyls of the blend [31]. The peak at 2900 cm^−1^ and 1375 cm^−1^ corresponds to the C–H stretching and bending frequencies respectively. The characteristic peaks at 1440 cm^−1^ and 1130 cm^−1^ corresponding to the bonds B–O and B–O–C [34], which proves the crosslinking reaction as per Figure 1. Absence of peak at 1198 cm^−1^ confirms that absence of unreacted boric acid [34,35]. The peaks at 1580 cm^−1^ and 1800 cm^−1^ shows the presence of aromatic rings of tannin. Overall, by the introduction of boric acid to PVA/tannin blend, FTIR spectra showed the confirmation of structural changes.

### 3.3 Viscosity and pH

Table 2 shows the results of viscosity and pH of the blended samples. It is observed that with the increase in the concentration of boric acid, the viscosity increases. Viscosity data is in accordance to the observation made from FTIR spectra. The acidic condition provided by the blend favours the crosslinking reaction as per Figure 1. Due to the formation of crosslinks, a network structure is formed which consolidates the chain and restricts the chain mobility [36]. Hence as a result of reduction in chain mobility, the sample becomes more viscous [37]. In addition to this, as concentration of boric acid is doubled from 0.5 wt.% to 1 wt.%, the sample becomes highly viscous. The increase in viscosity is advantageous from the point of wood adhesive since an optimum range is needed for proper spreading over the wood substrate. Since a very low or a very high viscous adhesive will have problem in wetting the wood substrate that can cause poor adhesion [30]. Overall, by introducing boric acid to PVA/tannin blend, increase in viscosity is observed.

**Table 2.** Results obtained from various analysis

Sample name	Boric acid (wt.%)	Viscosity (cps)	pH	Glass transition temperature (°C)	Pencil hardness value	Ultimate stress of film	Wet tack (gf)
Tan P blank	0	240 ± 12	4.6 ± 0. 1	86.21	2 H	33 ± 1.4	2269 ± 18
Tan P B 0.5%	0.5	540 ± 16	4.5 ± 0.3	91.80	4 H	47.2 ± 3.2	2354 ± 22
Tan P B 1%	0.1	1620 ± 24	4.4 ± 0.2	100.32	7 H	62 ± 5.4	2412 ± 14

### 3.4 Contact angle study

**Figure 3.**
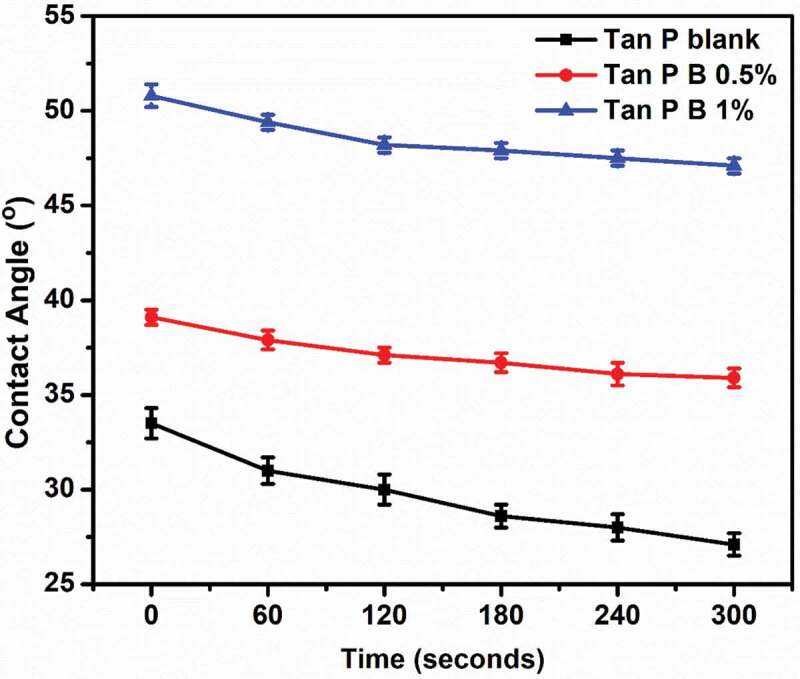
Water contact angle data with respect to time

To correlate with the hydrophilic character of modified blends with the blank sample, water contact angle measurement was studied. Distilled water is made in contact with the sample casted over glass slides and contact angle values were noted after each interval. Figure 3 shows the time-based contact angle of water with respect to the sample films. It is observed that the contact angle values are higher for crosslinked blends, than the blank sample irrespective of time. As the crosslinking occurs, free hydroxyl groups of PVA and tannin, due to the unavailable hydrophilic hydroxyl groups the crosslinked blends show less hydrophilic nature. And as the concentration of boric acid is doubled from 0.5 wt.% to 1 wt.%, more crosslinks are created on the blends and hence Tan P B 1% shows highest contact angle value throughout the experiment. The observation from FTIR that the reduction of hydroxyl groups in crosslinked samples provides an evidence for the same observation. Overall, the crosslinked blends exhibited more hydrophobic character which can be advantageous that the adhesive will have less influence with water giving a water-resistive property.

### 3.5 Differential scanning calorimetry (DSC)

**Figure 4.**
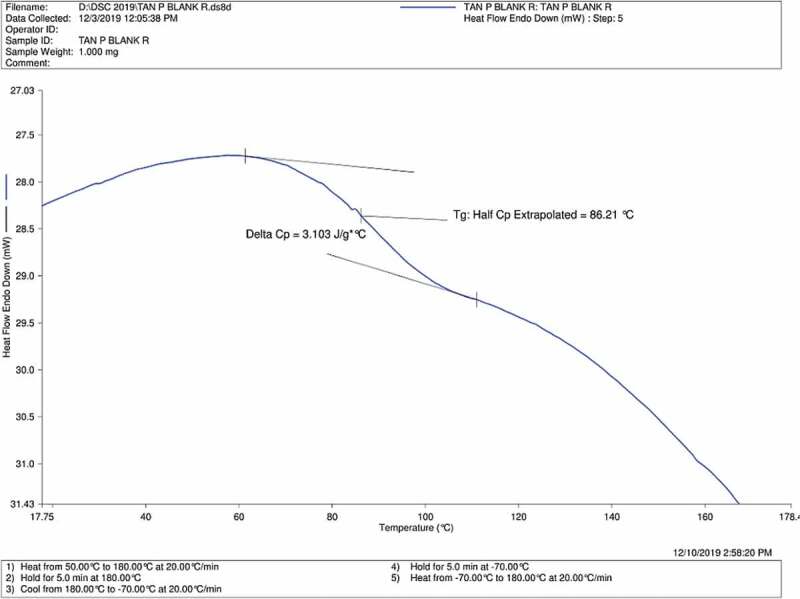
DSC profile of Tan P blank sample

**Figure 5.**
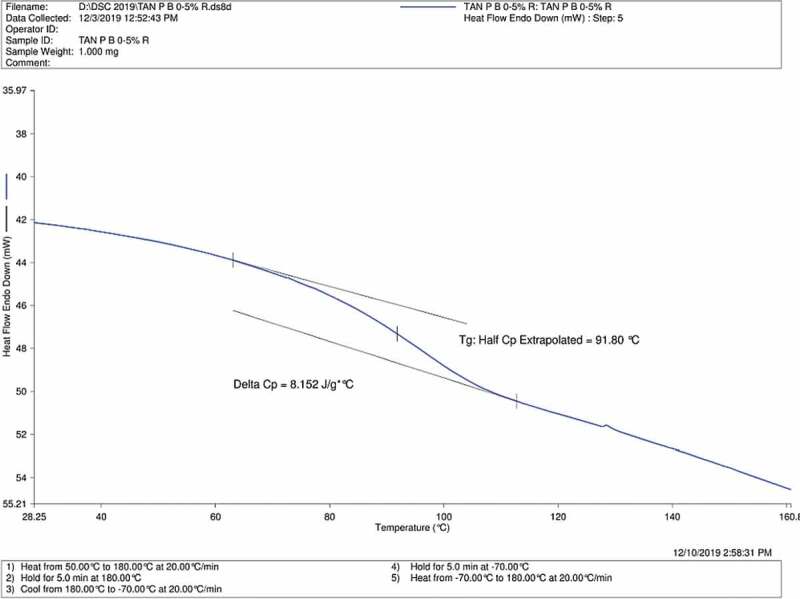
DSC profile of Tan P B 0.5% sample

**Figure 6.**
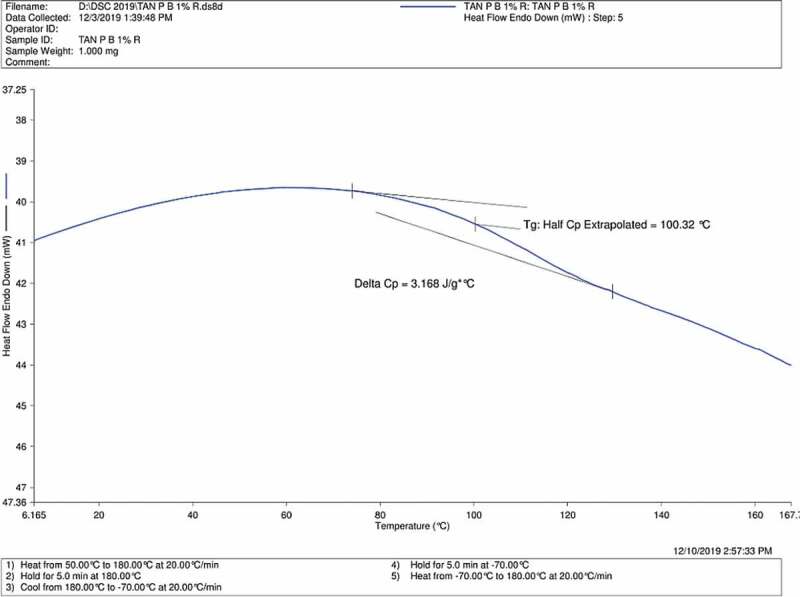
DSC profile of Tan P B 1% sample

Glass transition temperature (Tg) is an important parameter which relates to the thermo-physical transitions for the crosslinked blends [30]. Hence, Tg obtained from DSC results of Tan P blank, Tan P B 0.5% and Tan P B 1% are shown in Figures 4, 5 and 6 respectively. As the test film samples were properly dried before the DSC analysis, there is absence of water in the result. The results indicate that compared to the blank sample having Tg of 86.21°C, the crosslinked sample showed 91.8 and 100.32°C for samples with 0.5 and 1 wt.% boric acid respectively. Acidic condition favoured the crosslinking reaction of boric acid with the hydroxyl groups of PVA/tannin blend. The so formed crosslinks increased the chain length of the tannin and PVA while decreasing the void spaces between the chains, thus a reduction in chain mobility occurs and results in rise of Tg. As noticeable from the FTIR curves, there is a decrease in hydroxyl group of the adhesive with a subsequent increase in crosslinker. A similar trend is observed for Tg; there is an increase in Tg with an increase in the concentration of crosslinker. Hence, by DSC analysis, it can be concluded that enhancement in thermal properties with an increase in Tg is observed as the concentration of boric acid increases.

### 3.6 Pencil hardness test

The result obtained from pencil hardness test is shown in Table 2. The pencil hardness values of a film depend on the chemical structure of molecules and its flexibility. Tannin contains aromatic rings that are stiff which makes the lead pencils difficult to penetrate, thus contributes to the hardness of the film. When the chemical bonds are created by crosslinking reaction, further consolidation takes place. This causes reduction in free volumes and restricts the chain mobility as evidenced from decreasing peak intensity of hydroxy groups from FTIR and increase in Tg from DSC results. Hence crosslinking has positively enhanced the hardness of the films.

### 3.7 Ultimate stress of films

Ultimate stress of films crosslinked with boric acid is observed to have a positive shift towards the upper side from the results shown in Table 2. As evidenced from pencil hardness values, aromatic rings of tannin and the reduced free volumes between PVA and tannin contributed to this observation. And upon increasing the concentration of boric acid in the blend causes a proportional shift in its stress-bearing capacity, thereby increasing the stress required to break the film.

### 3.8 Wet tack analysis

To study the tackiness of adhesive wet tack test is performed. For better adhesion, the adhesive must have both adhesive forces and cohesive force. Result shown in Table 2 suggests that introducing boric acid to PVA/tannin blend contributed to enhancement in the wet tack property. As crosslinked structure reduces the mobility of polymer chains by consolidation, thereby enhances internal cohesive forces between the chains. Similar observations are seen from viscosity analysis, DSC and mechanical properties of the films. As more crosslinks are created on doubling up the boric acid content from 0.5 to 1 wt.%, Tan P B 1% showed maximum value of wet tack.

### 3.9 Tensile strength

**Figure 7.**
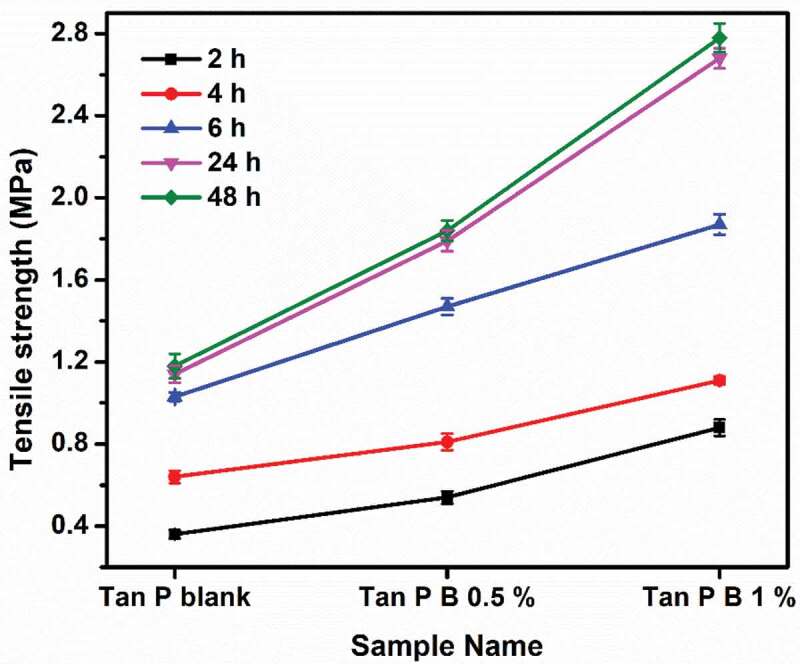
Tensile strength of canarium to canarium (can-can) wood substrate at various intervals

**Figure 8.**
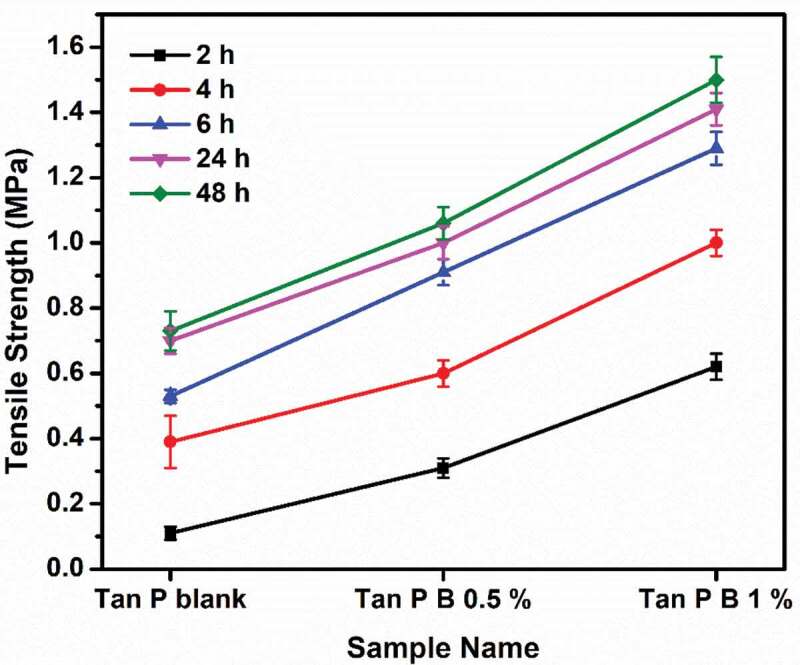
Tensile strength of plywood to laminate (ply-lam) substrate at various intervals

For understanding the performance property of the samples with respect to adhesion, tensile lap shear strength of bonded wood specimen is tested after particular time intervals [30]. Generally, in any water-based adhesives or coatings, curing takes place by the removal of water. The water in this case is removed by mainly three methods, (i) penetration of water from adhesive to wood substrate, (ii) evaporation of water from the adhesive, and (iii) since the crosslinked adhesive samples showed hydrophobic nature than the blank sample, it shows more tendency to repel water than the blank sample without boric acid. In this way the water is removed from the adhesive and hence bonding occurs. It can be noted that due to the presence of common hydroxyl groups in cellulose (wooden samples), PVA and tannin, it is reported that better bond formation occurs [38,39]. Figure 7 shows the tensile strength of bonded canarium to canarium (can-can) wood species while Figure 8 shows the tensile strength of bonded plywood to laminate (ply-lam) wood specimen. Can-can result gives idea on the hardwood behaviour of adhesives and the ply-lam corresponds to the hardwood (plywood) to softwood (laminate) type behaviour [40]. It is observed that in both the cases as boric acid content is increased tensile shear strength also increases, producing better adhesion to the substrates. The results of wet tack study in Table 2 support this observation. Enhancement in cohesive forces due to the crosslinks formed between polymer chain contributes to enhanced tensile strength. Increase in the tensile strength value with time denotes the time required for complete adhesion with the substrate. There is a not much increment in the rate of bond strength from 24 h to 48 h, revealing that almost complete bond formation occurs between 6 and 24 h. Care should be taken while handling the samples bonded at 2 h, since the bond development is very low. Lower value of tensile strength in ply-lam substrate is due to the dissimilarities in substrates as laminate contains many pores where the wetting of adhesive is faster compared to the plywood [40]. Whereas in can- can bonding the similarity of wood substrate (hardwood) makes the adhesive to equally wet both the substrates hence better bonding is observed from Figure 7. In both the cases, increment in the values of tensile strength corresponding to the performance of adhesive was observed maximum at 6 h. This property of the adhesive can be advantageous especially for high tack and quick grab adhesives, where secondary operations can be made in woodworking industries. Overall, by crosslinking with boric acid, PVA/tannin blend showed enhancement in bonding strength on ply-lam and can-can wood substrates.

## 4 Conclusion

The crosslinking reaction of boric acid with PVA/tannin was confirmed by studying the chemical interactions from the peaks obtained from FTIR spectroscopy. Increase in viscosity was observed when boric acid was incorporated at different concentrations. The crosslinked films exhibited less hydrophilic character and maintained stable water contact angle with respect to time. Crosslinker has brought enhancement in thermal property of the blend indicated by a rise in Tg with increased concentration of boric acid. It was noted that the mechanical properties like pencil hardness value and ultimate stress of crosslinked films showed a shift in positive side. Performance properties like wet tack and tensile lap shear strength on can-can and ply-lam wood substrate proved the applicability of crosslinked blends as wood adhesive. It could be concluded that PVA/tannin blends crosslinked with 1 wt.% boric acid showed maximum wet tack property and tensile strength on bonded wood substrates with better viscosity, stable water contact angle, increment in Tg and mechanical properties which can be a potential in the further development of bio-based wood adhesive.
